# Metal nanoparticles as effective promotors for Maize production

**DOI:** 10.1038/s41598-019-50265-2

**Published:** 2019-09-26

**Authors:** Son A. Hoang, Liem Q. Nguyen, Nhung H. Nguyen, Chi Q. Tran, Dong V. Nguyen, Quy N. Vu, Chi M. Phan

**Affiliations:** 1 Institute of Materials Science, Vietnam Academy of Science and Technology, 18 Hoang Quoc Viet Street, Cau Giay, Ha Noi Vietnam; 2 Agricultural Genetics Institute, TuLiem, Ha Noi Vietnam; 3 Maize Research Institute, Dan Phuong, Ha Noi Vietnam; 4 Department of Chemical Engineering and Curtin Institute of Functional Molecules and Interfaces, Curtin University, Perth, WA6045 Australia

**Keywords:** Enzymes, Plant physiology

## Abstract

Zero-valent metal nanoparticles (Cu, Fe and Co) were prepared by the reactive method from their oxide with hydrogen. The energy-rich solutions of metal nanoparticles were used for treatment Maize seeds prior to sowing. The treatment significantly improved the germination rate and early growth. Furthermore, both SOD and APX enzyme activity in leaves were improved, and enhanced the metabolism of superoxide, leading to increased drought resistance. The method was applied to the field over three seasons and greatly improved the harvest. In particular, the implementation of Cu particles at 4 mg/kg increased the productivity of the two Maize species more than 20%.

## Introduction

Maize has been domesticated for more than 10,000 years^1,2^ and remains a key staple for many regions. Maize can tolerate low moisture conditions and low nutrient soil, and thus becomes strategic staple for arid regions, where agricultural farming relies entirely on rainwater. Recent decades, the presowing seed treatment has been widely studied for crops and vegetables, and increasingly used as an indispensable and practical technique to improve agricultural production. While chemical promoters have been proposed^3^, these are expensive and difficult to apply in these unfavourable conditions.

It has well-accepted that metal ions can penentrate the plant cell walls and interact with biological processes at a molecular level^4^. Nanoparticles, however, cannot peneatrate the plant easily due to the limiting pore size on the cell wall. As the pore size of plant cell walls is reportedly around 4 to 6 nm^5^, and thus larger than 10 nm nanoparticles should not be able to penetrate. On the other hand, it has been reported that metal nanoparticles can increase biological activities and plant growth^6^. The enhanced growth was evidenced with Silver^7^, Iron^8^, Copper^9,10^ and Zince Oxide^11^ nanoparticles. The metal nanoparticles, including Iron^12^ and Copper^10,13^, can release electron upon dissolving into water due to the hight reduction potential in water.The minuscule size can massively increase the specific surface area, up to 25 m^3^/g^12^, and thus increase energy release^14,15^ as well as a stable suspension. The increased surface area and thus catalytic effects of the nanoparticles are well reported in the literature^14,15^. Zero-valent metal nanoparticles with suitable redox potential energy provide enhanced photosynthetic process of plant by the electron transfer reactions (for instance Cu^0^/Cu^2+^and Fe^0^/Fe^3+^). The electron transfer rections concentrate protons inside the membrane vesicle and create an electric field across the photosynthetic membrane. In this process, the electron transfer reactions convert redox free energy into an electrochemical potential of protons. The energy stored in the proton electrochemical potential is used by a membrane bound protein complex (ATP-Synthase) to covalently attach a phosphate group to adenosine diphosphate (ADP), forming adenosine triphosphate (ATP). In addition, in the photosynthetic process, much of the energy initially provided by light energy is stored as redox free energy and to be used later in the reduction of carbon dioxide.

This study will examine the potential application of nanoparticles to maize plantations. In addition to Iron and Copper, Cobalt nanoparticles were also studied. The particles are conveniently applied during the soaking process, which is commonly applied before maize planting. After synthesis and characterization, the particles were suspended and sonicated to produce a warm colloidal solution for soaking. The seeds were then planted according a normal procedure. The plants were analysed during the first few weeks in the controlled environment as well as in the field. The effects of nanoparticles during the growth in controlled experiments were quantified weekly by measuring growth rate, chlorophyll and anthocyanin content. Furthermore, the effect on applied metals on the drought resistance, which is a critical factor for remote and mountainous area, was also evaluated. Once the optimal conditions were determined in the controlled conditions, the process is applied to maize farm in a mountainous region. The field application, of three metals, was repeated over three cropping seasons. The impact of metals are compared the controlled samples.

## Metal Nanoparticles Preparation and Pre-Sowing Maize Seeds Treatment

Three metals, Iron, Copper and Cobalt, were synthesized via reduction method^12^ at temperature 300–400 °C, a reduction time of 90 minutes, a hydrogen flow rateat 350 ml/min (described in the Methods), with a final size at between 30 and 70 nm, the purity of produce is great than 99.6%. After the preliminary study, it was found that concentration less 5 mg/L was sufficient to enhance the seeds germination. Similar concentration range was also reported for Iron^7^ and Copper nanoparticles^9,10^. Consequently, the concentration range between 3 and 5 mg/L was employed in this study. To optimize the sonication time for metal particles, z-potential was measured as a function of time.

**Figure 1:**
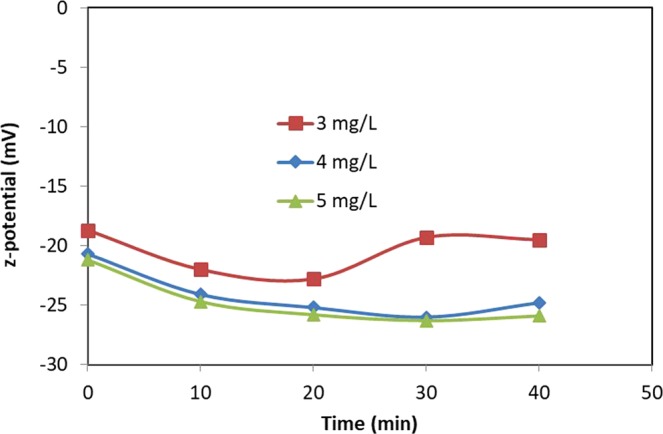
Zeta-potential of suspended Cu particles as a function of sonication time.

The data suggested that the release rate of electrons, due oxidation, reached a steady rate at approximately 20 minutes sonication. The weaker release at the shorter sonication time can be attributed to the partial dispersion of metal nanoparticles. From the *z-*potential data (Fig. 1), it has been established that 4 mg/L and 20 minutes sonication is the optimal conditions to apply the soaking process. The solution was immediately used in the soaking stage of seeds for 10 hrs. For each solution, 1 L was used to soak 1 kg Maize seeds, which was applied to 1000 m^2^.

## Early Growth

### Germination and early growth rate

The nano-metals were applied to a common Maize species obtained from Maize Research Institute (LVN-10). The samples were grown in a controlled environment for three weeks to ensure the consistency. The growth rates were measured via plant mass, root length, chlorophyll and anthocyanin content. Specifically, fresh and dry masses of a plant were recorded at the end of first week, second week and third week after the planting. The chlorophyll, anthocyanin content and the root length were measured after 3 weeks. Five samples were selected for each analysis to get average values and deviations.

**Figure 2:**
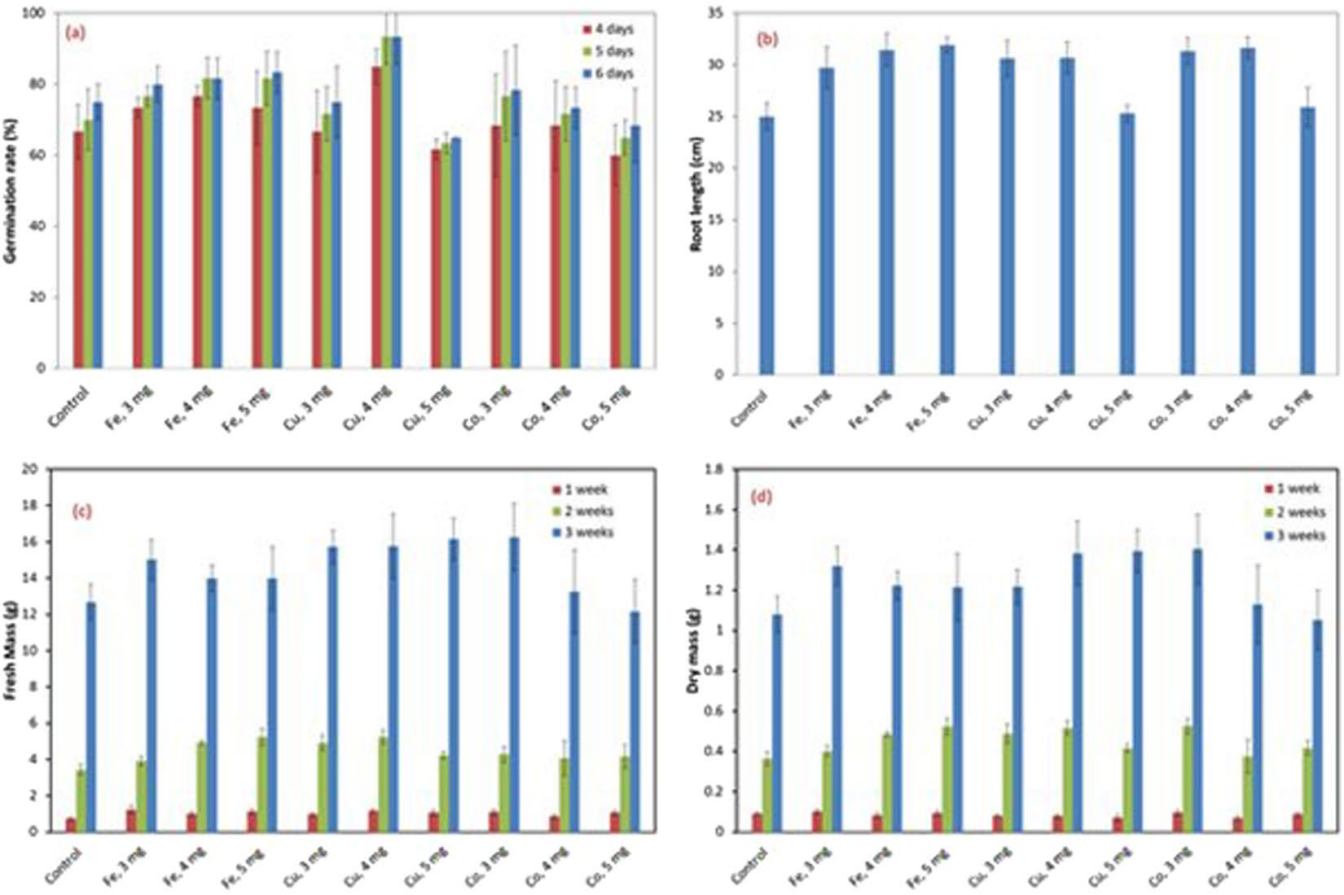
Germination rates (**a**). Root length (**b**), total mass (**c**) and dry mass (**d**) of the corn plant in the first three weeks.

In Fig. 2a, it can be seen that the Iron increased the germination rate significantly. Cobalt, on contrast, reduced the germination rate. Copper, at higher concentrations, reduced the germination rate. The root development after 3 weeks (Fig. 2b), was also corresponding to the germination.

The fresh and dry masses (Fig. 2) indicate that Iron and Copper particles have a positive impact on the plant growth. However, Cobalt, especially at high concentrations, actually reduced the obtained mass.Chlorophyll was monitored during the first three week to predict the strength of the seedlings. Anthocyanin, which is an important indication of plant health^16^, was also analysed during the same period.

**Figure 3:**
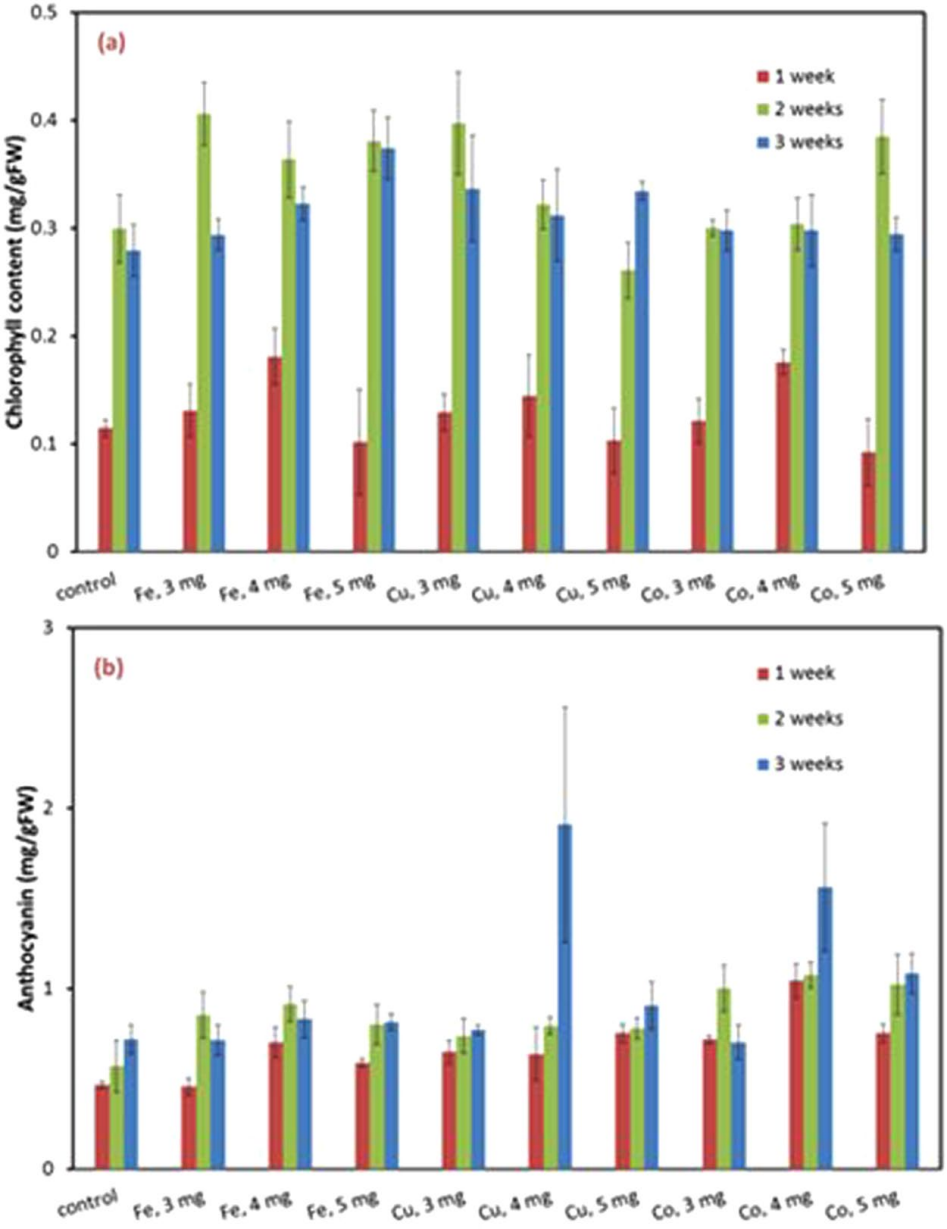
Chlorophyll (**a**) and Anthocyanin (**b**) content after the first three weeks.

It can be seen that chlorophyll was improved by metals. Furthermore, Cu significantly increased the anthocyanin (Fig. 3b). It is interesting to notice the selective impact of the metals. For instance, Cu and Co have promoted plant body better than Fe during the germination stage. On the other hand, Fe induced stronger rooting development than the other two (Fig. 3). Fe also produced highest chlorophyll content, whereas Cu and Co (4 mg/kg) produced higher anthocyanin content. The analysis in this stage indicated that Fe has the strongest effect the very early stage, within first week, in which the root development is the rate-determining factor. The impact of Iron nanoparticles was similar to reported observation with wheat shoots^8^. After two weeks, however, Cu and Co had stronger impacts. At this stage, body and leave development is more important, whereas the root system had fully established. The concentration of 4 mg/kg was selected for field application.

### Drought resistance and enzyme activity

**Figure 4:**
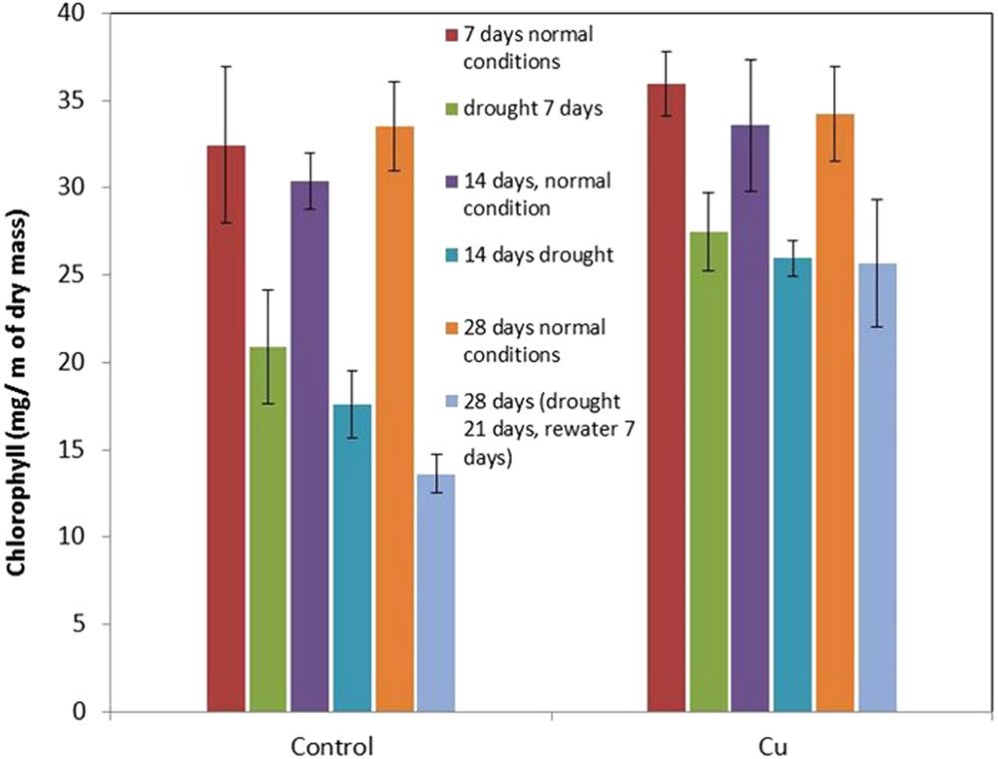
Chlorophyll content after drought conditions.

After the initial test, Cu at 4 mg/kg was considered the best application. Consequently, drought condition was articficially generated to investigate the impact of the Cu application. Evaluating some physiological and biochemical indicators at 7 days (slight term, corresponding to the relative water content in leaves decreased ~8–15%), 14 days (medium term, corresponding to Relative water content in leaves decreased by ~15–30%) and 21 days (heavy drought, corresponding to relative water content in leaves decreased by more than 30%) causing drought in artificial drought experiments. For the 21 days drought, the plants were re-watered again for 7 days. The chlorophyll contents were analysed (Fig. 4).

**Figure 5:**
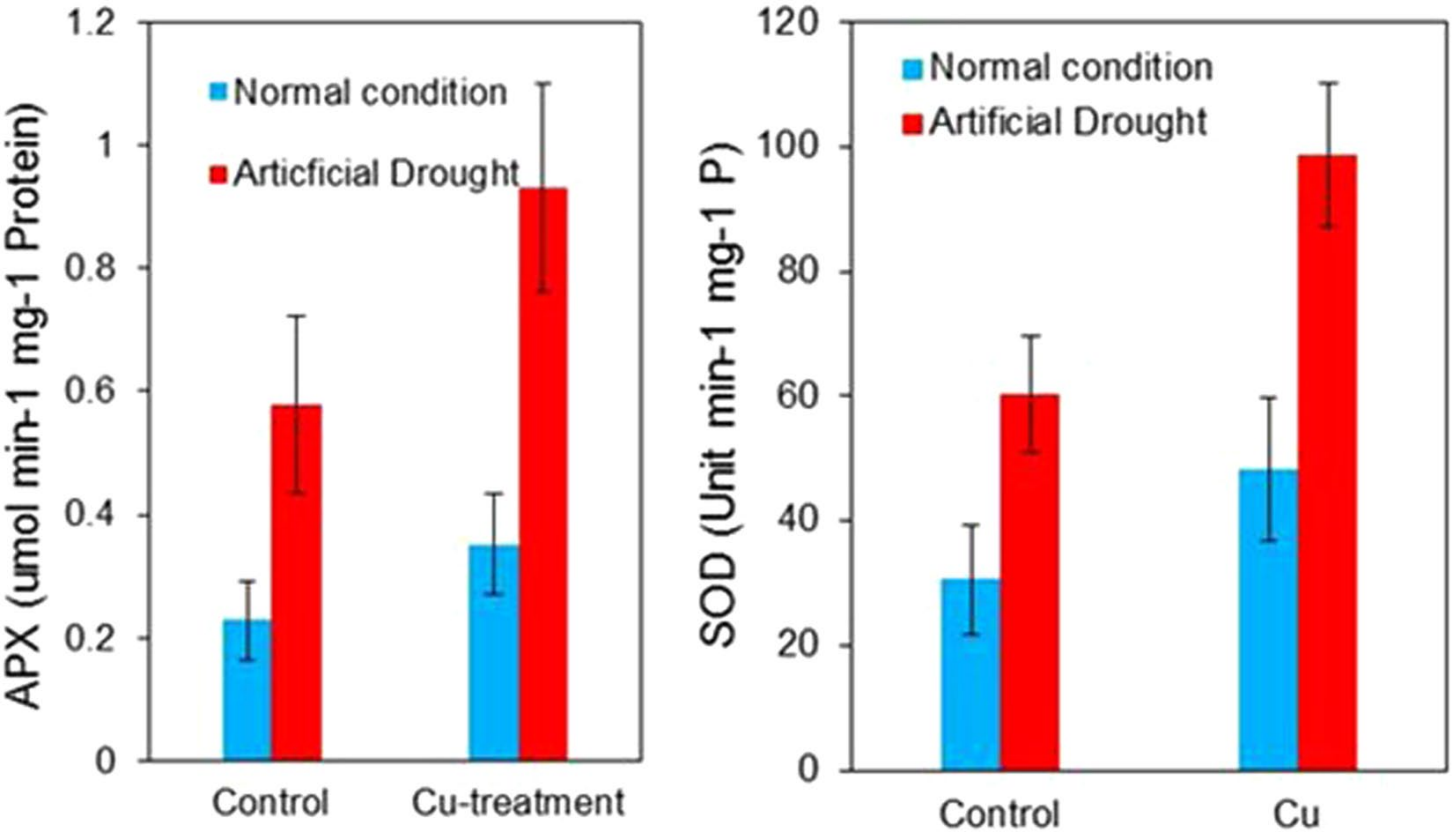
Enzyme analysis after 14 days drought (**a**) APX content, (**b**) SOD content.

The plants after the rewatering were compared in three important factors^17^: total protein, ascorbate peroxidase and superoxide dismutase. The total protein was measured by Bradford’s method^18^. Ascorbate peroxidase (APX)^19^ and superoxide dismutase (SOD) activity were estimated by standard methods^20^ and presented in Fig. 5.

It can be seen that Cu treatment dramatically increased the drought resistance of the plants. The ability to retain water is very critical for the field application. It is known that ion transport efficiency is highly dependent on the synthesis and hydrolysis of adenosine triphosphate (ATP) and electron transport^21^, which are the basic processes in plant cell activity during photosynthesis and respiratory. Respiration and associated electron transport depend directly on the substrates produced by dark-colored photosynthesis^22^. The release of free energy during respiration occurs in the mitochondria, in which the released energy is stored in ATP to provide energy for various biochemical processes^23^.

Compared with non-treated plants, maize treated with copper nanoparticles in drought conditions reduced by 30% of seeds (per plant) and decreased by 20% of seed weight (Table 1). Meanwhile, in control plants in drought conditions showed a decline of 77% of seeds (per plant) and 61% of seeds (per plant).

**Table 1:** Crop yield of Maize plant in normal and artificial drought condition.

Type	Condition	Total corn seeds/plant	Mass of corn/plant (gram)
Control	Normal	213,667 ± 29,948	35,167 ± 3,032
	Drought	71,000 ± 19,799	13,635 ± 4,137
Treated	Normal	216,333 ± 17,308	44,324 ± 3,629
	Drought	152,667 ± 25,486	35,290 ± 4,7128

**Figure 6:**
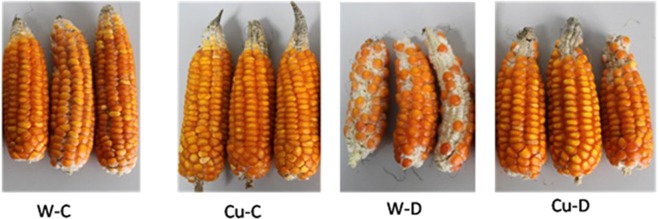
Corn of Maize plants was treated by nano Cu and control in normal and artificial conditions (W-C: Control in normal condition, Cu-C: Treated in normal condition, W-D: Control in drought condition, Cu-D: Treated in drought condition).

This result shows the potential of the application of Copper nanoparticles in maintaining corn yield in drought conditions. Results of grain yield were also consistent with the maintenance of higher chlorophyll content in corn treated with copper nanoparticles in drought conditions compared to the respective control plants. The improvement can be viasually observed as shown in Fig. 6.

## Field Application

The plants were carried at Mai Son District, Son La Province. The first season is from June to October, 2016 (autumn cropping season). Two species were employed obtained from Maize Research Institute, the LVN-10 and VN-8960.In addition to productivity, the quality of the harvest was also analysed. In particular, the length and diameter of the corn ears, the number and arrangement of rows within the ears and the specific mass of 1000 kernels.

The impact on the ear length was more distinguishable for VN8960 than for LVN10. Specifically, the Cu-enhancement produced the longest ear, at 17.3 cm. On the other hand, Co produced the shortest ear at 14.4 cm. The ear diameter varied slightly within 4.3 and 4.6 cm. The difference in term of row was insignificant. The specific mass (per 1000 kernels) increased slightly by the metal applications. For LVN10, in particular, Cu–enhanced sample has a specific mass of 334.3 g/1000 kernels, which was 4% higher than the controlled sample.

**Figure 7:**
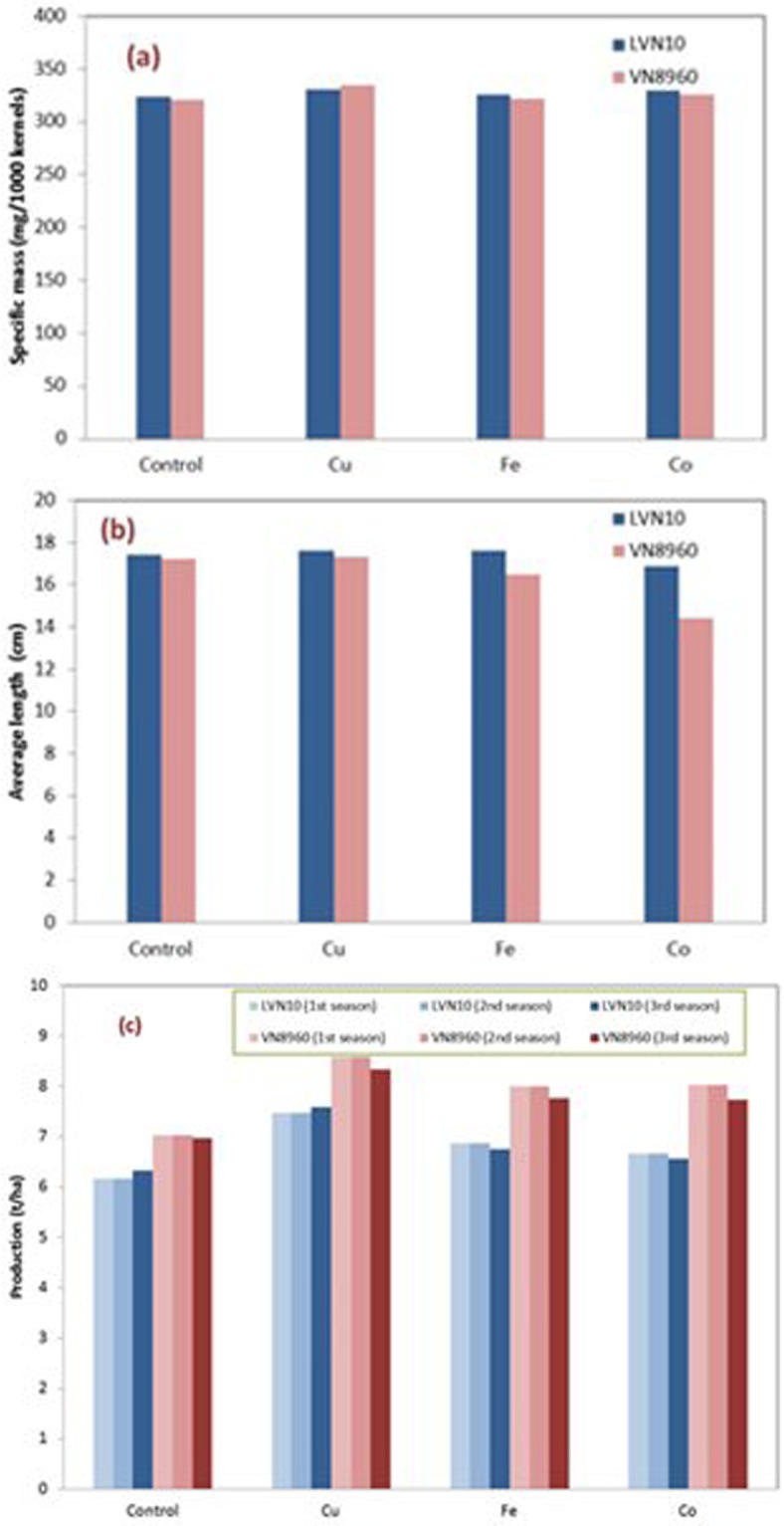
Quality of the harvest: (**a**) specific mass, (**b**) average length and (**c**) productivity.

Most importantly, the production for both species increased by more than 20% by Cu-enhancement. For VN8960, which had highest production, Cu increased the harvest to 8.589t/ha, against the controlled sample of 7.030 t/ha (Fig. 7). This represents 22% increment with a probability ≥95%. Similarly, the increased production was 21% for LVN10 with a probability ≥95%. The incensement was likely come from the increased number of ears, rather than the size of the corn earor specific mass (as indicated in Fig. 7). Amongst the three metals, Co produced the weakest improvement (less than 10%). Repeating application in the following two seasons, Spring 2017 (February to June) and Autumn 2017 (June to October), also demonstrated more than 20% increment over the controlled samples. Finally, the metal contents were analysed for all samples by ICP-AES. It was found that metal residues are statistically insignificant (Table 2) and far less than the safety limits recommended by WHO/FAO^24^.

**Table 2:** Determination of metal residues in harvested maize seeds.

	Metal concentration (mg/kg)		
	Cu	Fe	Co
Cu treatment	2.4	7.2	<1
Fe treatment	1.6	6.7	<1
Co treatment	3.1	8.4	<1
Control	2.7	7.8	<1
WHO safety limit	73.3	425.5	50

## Conclusion

It has been found that metal nanoparticles have greatly improved the growth and productivity of corn. The impact was clearly demonstrated throughout the plant development. Amongst the three metals, Cu has highest productivity, more than 20% for the two Maize species, over the controlled sample. The amount of the nanoparticles was very small, at 4 mg/kg in the soaking solution, and does not have any residual effects on the harvest. The increased production was consistent with increased chlorophyll content and drought resistance observed in the early growth stage. The results validatedan easy and economical pathway to improve corn productivity, especially for the rural areas without access to advanced facilities.

## Methods

The process of obtaining nano powders of iron, copper and cobalt includes three steps: (1) bbtaining hydroxide from salt, (2) precipitation of metal hydroxides from the solution of NaOH, and (3) dehydration of hydroxides to obtain their oxides. All precusors, CuSO_4_.5H_2_O, Fe(NO_3_)_3_.9H_2_O, Co(NO_3_)_2_.6H_2_O, NaOH and NH_4_OH were obtained from Sigma Aldrich. The metal oxides were obtained by reacting with NaOH. Consequently, metal oxides are formed. Metal oxides of Iron, Copper and Cobalt were prepared by the reduction reaction method using freshly hydrogen from hydrogen electrolysis as a reducing agent. The particles were analysed by SEM (Hitachi S-4800) and X-ray diffraction (XRD XRD 3100 diffract meter at 45 kV and 30 mA). The XRD pattern confirmed that an amourphous phase of Copper was formed after 90 minutes of reduction reaction.

Protein content was analysed by grinding leave sample in 1 ml of extraction buffer before adding to 10 ml of Bradford solution. The sample was then measured at wavelength of 595 nm. The Bradford reagent was prepared by mixing 0.02 g of CBB G-50, 10 mL ethanol, 20 mL phosphoric acid and 170 ml of di-ionized water. The protein standard curve was obtained by 10 mg of bovine serum albumin (BSA) and 10 ml di-ionized water. Ascorbate Peroxidase (APX) was measured by monitoring the decrease in absorbance at 290 nm as AsA was oxidized. Superoxide dismutase (SOD) activity was estimated by a xanthine–xanthine oxidase system. The reaction mixture contained K_3_PO_3_ buffer (50 mM), Nitrobluetetrazolium (2.24 mM), catalase (0.1 units), xanthine oxidase (0.1 units), xanthine (2.36 mM), and enzyme extract. SOD activity was expressed as units (i.e., amount of enzyme required to inhibit NBT reduction by 50%) per minute per milligram protein.

The field productivity and quality measurements are specified as:

- Corn length (cm): Measured in the longest grain of corn ear.
- Corn diameter (cm): measure at the middle of the corn ear.
- Number of seeds on corn: Count the number of items with more than 1 corn ear (1 row is counted when there is 50% of the number of seeds compared to the longest).
- Number of seeds /row: Counted by the average length of grain.
- Mass of 1000 seeds (gram): At the moisture content of 14%, take 2 samples, each with 500 seeds. The measurement is accepted if the difference between the two weighings does not differ by more than 5% from the average weight.
- Percentage of seeds /corn at harvest (%): Each formula takes an average of 10 representative corn samples in the plot, taking seeds to calculate the ratio.
- Humidity at harvest (%): Sampling as the ratio of seed / corn and measured by KETT - GRAINERII-400. Whereas the net yield (weight/ha) is calculated at a moisture content of 14%, after substracting the core mass.

## Supplementary information

Related Manuscript File
