# Author Correction: Metal nanoparticles as effective promotors for Maize production

**DOI:** 10.1038/s41598-021-81981-3

**Published:** 2021-01-26

**Authors:** Son A. Hoang, Liem Q. Nguyen, Nhung H. Nguyen, Chi Q. Tran, Dong V. Nguyen, Nga. T. Le, Chien. V. Ha, Quy N. Vu, Chi M. Phan

**Affiliations:** 1 Institute of Materials Science, Vietnam Academy of Science and Technology, 18 Hoang Quoc Viet Street, Cau Giay, Ha Noi, Vietnam; 2 Agricultural Genetics Institute, TuLiem, Ha Noi, Vietnam; 3 Maize Research Institute, Dan Phuong, Ha Noi, Vietnam; 4 Department of Chemical Engineering and Curtin Institute of Functional Molecules and Interfaces, Curtin University, Perth, WA 6045 Australia

Correction to: *Scientific Reports* https://doi.org/10.1038/s41598-019-50265-2, published online 26 September 2019

In the original version of this Article, Chien Ha and Nga Thanh Le were omitted from the author list. The complete author list, and affiliations, is listed below,

Son A. Hoang^1^*, Liem Q. Nguyen^1^, Nhung H. Nguyen^1^, Chi Q. Tran^1^, Dong V. Nguyen^2^, Nga. T. Le^2^, Chien. V. Ha^2^, Quy N. Vu^3^ and Chi M. Phan^4^

^1^Institute of Materials Science, Vietnam Academy of Science and Technology, 18 Hoang Quoc Viet Street, Cau Giay, Ha Noi, Vietnam

^2^Agricultural Genetics Institute, TuLiem, Ha Noi, Vietnam

^3^Maize Research Institute, Dan Phuong, Ha Noi, Vietnam

^4^Department of Chemical Engineering and Curtin Institute of Functional Molecules and Interfaces, Curtin University, Perth WA6045, Australia

As a result, the Author Contributions statement now reads:

“D.N., Q.V., N.L., C.H. reagents and analytic tools, performed research.”

In addition, the legend of Figure 5 contained an error:

“Enzyme analysis after 14 days drought (a) APX content, (b) SOD content”

and now reads:

“(a) APX and (b) SOD enzyme activity of maize leaf at 7 days under drought stress”

Finally, some methodological details were not provided in the original Article, and the following text has been added to the Supplementary Information file, under the subheading ‘3. Chlorophyll, Protein and Anthocyanin analysis’:

“The fresh plant mass was measured after washing the plants and gently placed on the filter paper. Subsequently, the dry weight was obtained after 48 h of drying in an oven at 65∘C. To determine the leaf relative water content, detached leaves were individually weighed to determine sample weight (W). Subsequently, the samples were fully dipped in deionized water overnight under normal room light and temperature for rehydration to full turgidity. The samples were then gently removed with filter paper and were immediately weighed to obtain a fully turgid weight (TW). Afterwards, the samples were dried in an oven at 65∘ C for 48 h, and dry weight was measured (DW). The relative water content (RWC) was calculated as RWC (%) = [(W − DW)/(TW − DW)]^1^.

For chlorophyll measurement, an amount of 0.5 g leaf sample was extracted in 20 mL of extraction solution (acetone 80%) by overnight shaking (200 rpm) in the dark condition at room temperature. After the extraction, 1 mL of extraction solution was used for measuring the absorbance of chlorophyll at 645 nm and 663 nm via the spectrophotometry (Thermo Scientific Gensys 20, USA). The total chlorophyll content was calculated based on the formula in the literature^2^.

For anthocyanin measurement, 0.02 g leaf was ground in liquid nitrogen and extracted by 300 µL of extraction solution (methanol plus 1% HCl) at 4 °C overnight. The solution was then mixed with 200 µL of water and 200 µL of chloroform. Consequently, the solution sample was centrifuged at 4 °C for 15 min (3000 rpm). The anthocyanin content was determined from the absorbance at 530 nm^3^.”

These errors have now been corrected in the PDF and HTML versions of the Article, and in the accompanying Supplementary information file.
