# Supplementary material for: Metal nanoparticles as effective promotors for Maize production
Source: Sci Rep. 2019 Sep 26;9:13925. doi: 10.1038/s41598-019-50265-2 (PMC6763462; doi:10.1038/s41598-019-50265-2)
Supplement: Supplementary file 1 — Related Manuscript File [file 41598_2019_50265_MOESM1_ESM.docx]

**Metal nanoparticles as effective promotors for Maize production**

Son A. Hoang^1^, Liem Q. Nguyen^1^, Nhung H. Nguyen^1^, Chi Q. Tran^1^, Dong V. Nguyen^2^, Quy N. Vu^3^ and Chi M. Phan^4^

^1^Institute of Materials Science, Vietnam Academy of Science and Technology, 18 Hoang Quoc Viet Street, Cau Giay, Ha Noi, Vietnam

^2^Agricultural Genetics Institute, TuLiem, Ha Noi, Vietnam

^3^Maize Research Institute, Dan Phuong, Ha Noi, Vietnam

^4^Department of Chemical Engineering and Curtin Institute of Functional Molecules and Interfaces, Curtin University, Perth WA6045, Australia

# Supporting Information

## 1. Particles Synthesis

The process of obtaining nano powders of iron, copper and cobalt includes the following main stages:

- Obtaining hydroxide from salt Cu(NO_3_)_2_.H_2_O; Fe(NO_3_)_3_.9H_2_O; Co(NO_3_)_2_.6H_2_O
- Precipitation of Fe(OH)_3_; Cu(OH)_2_; Co(OH)_2_ from the solution of NaOH
- Dehydration of hydroxides to obtain their oxides

Cu(NO_3_)_2_.H_2_O, CuSO_4_.5H_2_O, Fe(NO_3_)_3_.9H_2_O, Co(NO_3_)_2_.6H_2_O, NaOH, NH_4_OH were obtained from Sigma Aldrich. The metal oxides were obtained by reacting with NaOH. Consequently, metal oxides are formed. Nano metals of Iron, Copper and Cobalt were prepared by the reduction reaction method using freshly hydrogen from hydrogen electrolysis as a reducing agent.

## 2. Particles Characterization

### Scanning Electron Microscopy and Dynamic Light Scattering

The surface morphology of zero –valent copper nanoparticle was revealed by SEM (Hitachi S-4800) picture (Figure 8). The size of copper nanoparticle was about 30-40 nm.

**
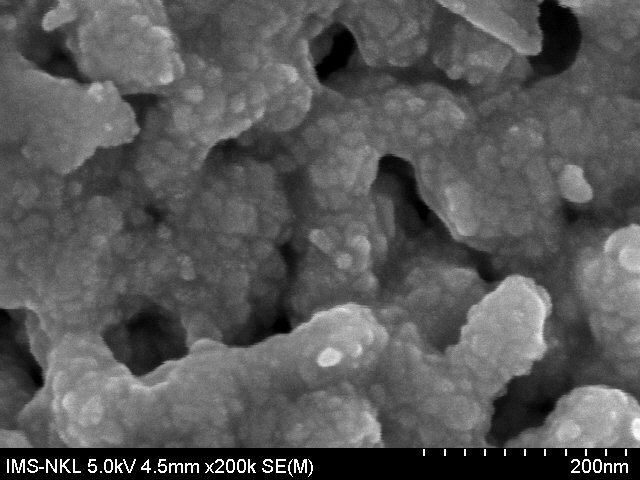

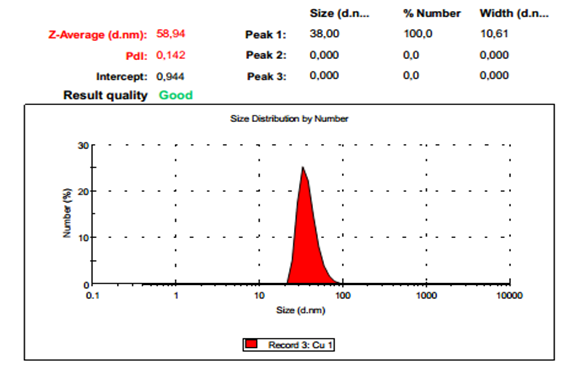
**

Figure S1. SEM image and Zeta size analys of zero–valent copper nanoparticle

An accumulative size distribution survey of Zetasize analyse suggests that over most of the nanoparticles have an average hydrodynamic diameter of 58.94 nm whereas 90% were within 25 – 50 nm.

### X-ray diffract meter

The method of X-ray diffraction (XRD) was used to investigate the material structure of copper nanoparticles. The XRD analysis was conducted with a XRD 3100 diffract meter at 45 kV and 30 mA. It used copper Kα radiation and graphite monochromatic to produce X-rays with a wavelength of 1.54060 Å. Copper nanoparticles were placed in a glass holder and scanned from 20° to 90°. This scan range covered all major species of iron and iron oxides. The scanning rate was set at 2.0°/min. The characteristic broad peak at 2*θ* of 45º indicated that the zero valent copper was predominantly present in the sample.

Here it was seen that the diffract grams is same and good agreement with the XRD pattern of iron nanoparticles. On the figure 9, it revealed that the initial sample have structure of CuO. When the reaction occurs during 40 mins, part of nano copper particle was formed. By making the reaction during 90min, the broad peak at 43.38, 50.48 and 74.18 revealed the existence of an amorphous phase of copper. From the XRD pattern, the particle size of the copper nanoparticles was found to be 30-70 nm and the percentage of iron nanoparticles was more than 99.6% by weight. It should be noted that the required time (more 1 hour) for completing the reduction process was consistent with the reported time in the literature.

Figure S2. XRD pattern of (a) CuO precursor, (b) the reaction temperature 200^0^C, (c) the reaction temperature 300^0^C

Figure S3. XRD pattern (a) reaction time 10 min, (b) reaction time 20 min

## 3. Chlorophyll, Protein and Anthocyaninanalysis

The sample was grinded in the 1ml of extraction buffer before adding to 10 ml of Bradford solution. The sample was then measured at wavelength of 595 nm. The Bradford reagent was prepared by mixing 0.02 g of CBB G-50, 10 mL ethanol, 20 mL phosphoric acid and 170 ml of di-ionized water. The protein standard curve was obtained by 10 mg of bovine serum albumin (BSA) and 10ml di-ionized water.

Ascorbate Peroxidase (APX) was measured by monitoring the decrease in absorbance at 290 nm as AsA was oxidized. Superoxide dismutase (SOD) activity was estimated by a xanthine–xanthine oxidase system (Figure 11). The reaction mixture contained K_3_PO_3_ buffer (50 mM), Nitrobluetetrazolium (2.24 mM), catalase (0.1 units), xanthine oxidase (0.1 units), xanthine (2.36 mM), and enzyme extract. SOD activity was expressed as units (i.e., amount of enzyme required to inhibit NBT reduction by 50 %) per minute per milligram protein.

**4. Production testing methods and evaluation of the components of capacity and productivity**

Conducting experiments "according to the method of production testing", the experiment consists of 10 formulas (9 formulas processed by 3 types of nano metal at 3 different concentrations and 1 unprocessed formula), each formula 3 times repeated.

Table S1. Diagram of a random design model with 10 formulas and 3 replicates

| **TT** | **Formula** | **Repicates** | | |
| --- | --- | --- | --- | --- |
|  |  | **I** | **II** | **III** |
| 1 | CT1 | 1 | 14 | 25 |
| 2 | CT2 | 2 | 16 | 22 |
| 3 | CT3 | 3 | 11 | 26 |
| 4 | CT4 | 4 | 19 | 30 |
| 5 | CT5 | 5 | 12 | 23 |
| 6 | CT6 | 6 | 18 | 29 |
| 7 | CT7 | 7 | 13 | 21 |
| 8 | CT8 | 8 | 20 | 28 |
| 9 | CT9 | 9 | 15 | 24 |
| 10 | CT10 | 10 | 17 | 27 |

| CT1: | Control | CT6: | Nano Co^0^ - 4 mg/kg |
| --- | --- | --- | --- |
| CT2: | Nano Cu^0^ - 3 mg/kg | CT7: | Nano Co^0^ - 5 mg/kg |
| CT3: | Nano Cu^0^ - 4 mg/kg | CT8: | Nano Fe^0^ - 3 mg/kg |
| CT4: | Nano Cu^0^ - 5 mg/kg | CT9: | Nano Fe^0^- 4 mg/kg |
| CT5: | Nano Co^0^ - 3 mg/kg | CT10: | Nano Fe^0^- 5 mg/kg |

The components of productivity and productivity:

- Corn length (cm): Measured in the longest grain of corn.

- Corn diameter (cm): Measure the middle of the corn.

- Number of seeds on corn: Count the number of items with more than 1 corn (1 row is counted when there is 50% of the number of seeds compared to the longest).

- Number of seeds /row: Counted by the average length of grain.

- Mass of 1000 seeds (gram): At the moisture content of 14%, take 2 samples, each with 500 seeds. If the difference between the two weighings does not differ by more than 5% from the average weight of the two samples, it is okay.

- Percentage of seeds /corn at harvest (%): Each formula takes an average of 10 representative corn samples in the plot, taking seeds to calculate the ratio.

- Humidity at harvest (%): Sampling as the ratio of seed / corn and measured by KETT - GRAINERII-400

- Net yield (weight/ha) at moisture content of 14% is calculated according to the formula:

FW x (P1 - P2) x (100 - MC) x 100

Net yield (ton/ha) = ------------------------------------------

S x P1 x (100 - RC)

Where: FW: Mass experiment plot (kg) MC: Humidity at harvest (%)

P1: Sample volume mẫu (gam) P2: Core volume (gam)

RC: Standard moisture grain corn (14 %) S: Area of experiment plot (m^2^)
